# Trends in prescription therapy for adolescents with depression in nine major areas of China during 2017–2021

**DOI:** 10.3389/fpsyt.2023.1175002

**Published:** 2023-10-26

**Authors:** Li Wang, Linpo Zhou, Yao Zhu, Jingjing Yan, Na Bu, Weidong Fei, Fan Wu

**Affiliations:** ^1^Department of Pharmacy, Women’s Hospital, Zhejiang University School of Medicine, Hangzhou, China; ^2^The Fourth School of Clinical Medicine, Zhejiang Chinese Medical University, Hangzhou, China; ^3^Department of Pharmacy, The First Affiliated Hospital of Zhejiang Chinese Medical University, Zhejiang Provincial Hospital of Chinese Medicine, Hangzhou, China

**Keywords:** adolescent depression, antidepressant, antipsychotic, sertraline, prescription

## Abstract

**Objective:**

To date, no national-scale drug usage survey for adolescents with depression has been conducted in China, and the purpose of this study was to examine the national trends in prescriptions in Chinese adolescent depression patients from 2017 to 2021.

**Methods:**

Prescribing data were extracted from the Hospital Prescription Analysis Cooperative Project. The average number of patients per year, the cost of treatment, and the prescription patterns (monotherapy vs. combination therapy) were analyzed, and subgroup analyses were conducted depending on age, sex, and drug class.

**Results:**

The study included 674,099 patients from 136 hospitals located in nine major areas of China. Of all patients, the proportion of adolescents increased from 1.63% in 2017 to 6.75% in 2021. Visits by adolescent depression patients increased from 1,973 in 2017 to 9,751 in 2021, and the corresponding cost increased from 607,598 Chinese Yuan in 2017 to 2,228,884 Chinese Yuan in 2021. The incidence of adolescent depression among female individuals was far beyond that among male individuals. Combination therapy was more frequent than monotherapy, and the most commonly prescribed drugs were antidepressants, antipsychotics, antiepileptics, and antianxietics. Despite the use of sertraline decreasing from 47.90 to 43.39%, it was the most frequently used drug.

**Conclusion:**

In summary, the prescriptions and cost of treatment for adolescent depression patients both increased rapidly. The widespread use of those drugs with weak clinical evidence reflects the current state of China, which should arouse our attention. The study can provide references for clinical treatment decisions and a basis for more efficient allocation of healthcare resources by the government.

## Introduction

Depression is one of the most common mental disorders that seriously affects the patients’ psychosocial functions and quality of life. Adolescence is a key life period characterized by rapid social and emotional development, so it is a vulnerable time for depression (1). Despite all age groups experiencing an increase in depression prevalence, the rate of increase among adolescents was significantly more rapid relative to adults (2). In adolescents aged 13–18 years in the United States, the lifetime and 12-month prevalence of major depressive disorder (MDD) were 11.0 and 7.5%, respectively (3), according to the “China National Mental Health Development Report (2019–2020)” released by the Institute of Psychology of the Chinese Academy of Sciences. Detection rate is the detection rate of depressive symptoms. The survey was conducted using a self-rating scale. According to the score, there is no, mild, or severe depression. The results of the scale can only reflect the severity of depression at a certain time but not a diagnosis. The detection rate of depression among Chinese adolescents was 24.6%, and the rate of MDD was 7.4%. Adolescents with depression have an elevated risk of other psychopathology (4), physical health outcomes (5, 6) and recurrence in later life (7); meanwhile, the increase in suicide attempts, nicotine abuse, alcoholism, and drug abuse have been found to be associated with adolescent depression (8, 9).

Except for psychotherapy, drug therapy is one of the main treatment methods for depression in adolescents, and various factors influence the choice of drug, including the depression type, severity of the symptom, patient age, guidelines, prescribing doctor’s experience, cost, and so on. Therefore, understanding the current situation of adolescent depression drug usage is crucial for improving depression treatment. For now, there is little information available about the use of drugs for adolescent depression in China. Therefore, to assess the time trends and patterns of prescriptions for adolescents with depression, we conducted a cross-sectional study in nine major areas in China from 2017 to 2021.

## Methods

### Ethics

Ethics approval was obtained from the First Affiliated Hospital of Zhejiang Chinese Medical University for this study. This study was retrospective, so informed consent was not required.

### Data source

A database from the Hospital Prescription Analysis Cooperative Project was used to extract the data, and this approach has been used widely in Chinese pharmacoepidemiology studies and has been validated (10, 11). Participating hospitals provided prescription information for the database on sampling days. During the year, there were 40 randomized sampling days, not a full year of data; that is, the data were sampled randomly. The prescription data included the code, sex, age of patients, date, location, diagnosis, generic drug name, dose, and cost. This study included outpatient prescriptions meeting the following criteria: (1) prescriptions for patients aged 12–17 years; (2) those for the diagnosis of depression, without restrictions on the diagnostic criteria and severity of disease; (3) those issued from hospitals in Beijing, Shanghai, Guangzhou, Hangzhou, Chengdu, Zhengzhou, Shenyang, Tianjin, and Heilongjiang that participated in the program continuously from 2017 to 2021. Data were obtained from hospitals located in the east, west, south, and north of China, which covered a substantial geographical area and represented the entirety of the country.

### Analysis

Trends in proportions will be tested by the Cochran–Armitage trend test. Other trends will be analyzed by the Mann–Kendall trend test. The statistical analysis was conducted using R Version 4.2.1^1^ software.

## Results

### Total trends in visits and expenditure

**Figure 1:**
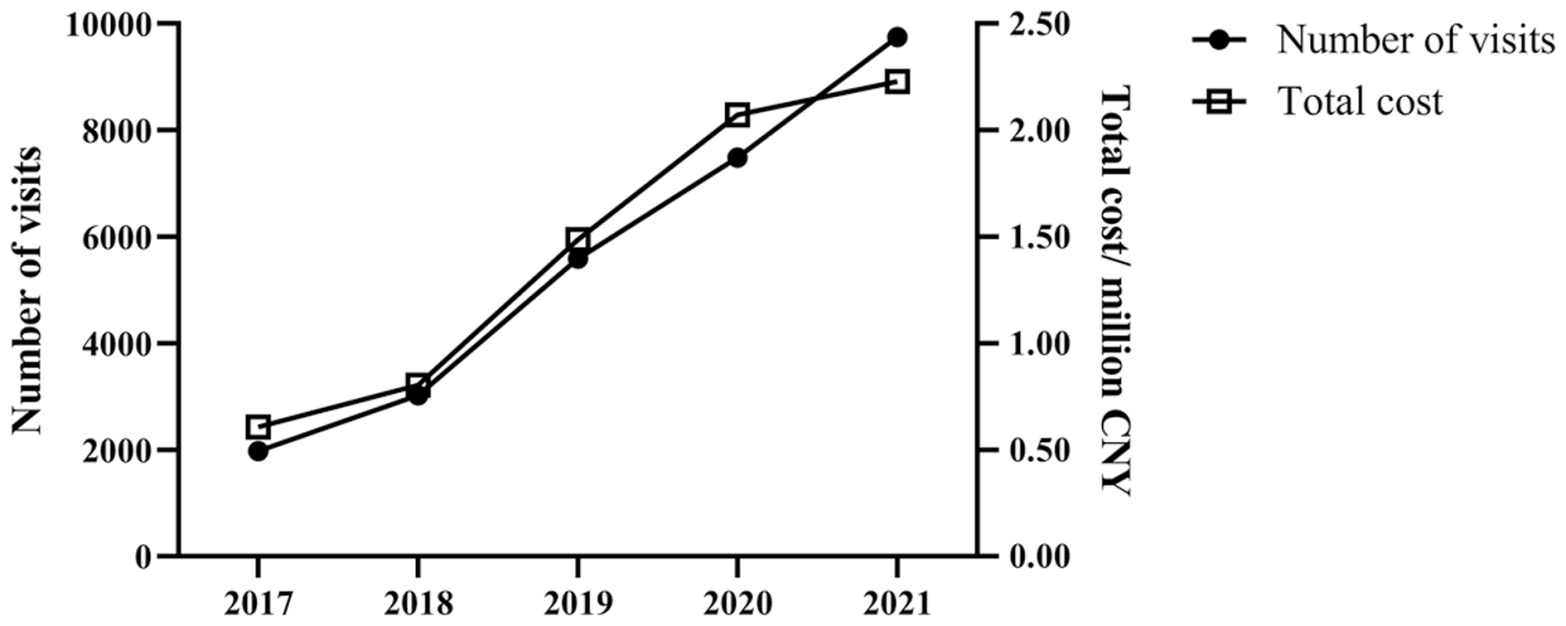
Trends in visits and cost of adolescents with depression in 136 hospitals located in nine major areas of China from 2017 to 2021.

A total of 674,099 patients issued from 2017 to 2021 were identified in this study. As Table 1 and Figure 1 indicate, during the study period, there was no significant trend in patients with depression (*p* = 0.462), while the proportion of adolescents increased from 1.63% in 2017 to 6.75% in 2021 (*p* < 0.001). The adolescence visits increased from 1,973 in 2017 to 9,751 in 2021 (*p* = 0.027), and the total expenditure increased dramatically from 607,598 Chinese Yuan (CNY) in 2017 to 2,228,884 CNY in 2021 (*p* = 0.027).

**Table 1:** Age structure of included patients, 2017–2021.

Age	Number of patients (%)					P_1_	P_2_
	2017	2018	2019	2020	2021		
12–17	1973 (1.63)	3,020 (2.21)	5,588 (3.85)	7,484 (5.91)	9,751 (6.75)	0.027	<0.001
≥18	119,346 (98.37)	133,780 (97.79)	139,449 (96.15)	119,066 (94.09)	134,642 (93.25)	0.807	<0.001
Total	121,319	136,800	145,037	126,550	144,393	0.462	

P_1_, *p*-value for trend in the number of patients, assessed by the Mann–Kendall trend test; P_2_, *p*-value for trend in the proportion of patients, assessed by the Cochran-Armitage trend analysis.

### Trends stratified by sex

A stratified analysis of the trends in depression among adolescents by sex is shown in Table 2, the proportion of females (67.56–76.68%) was generally higher than male patients (23.32–32.44%), and the proportion of females showed a significant increase trend, while the trend of males decreased obviously (both *p* < 0.001).

**Table 2:** Gender distribution of adolescent depressive patients, 2017–2021.

Sex	Number of patients (%)					P_1_	P_2_
	2017	2018	2019	2020	2021		
Male	640 (32.44)	957 (31.69)	1,447 (25.89)	1745 (23.32)	2,358 (24.18)	0.027	<0.001
Female	1,333 (67.56)	2063 (68.31)	4,141 (74.11)	5,739 (76.68)	7,393 (75.82)	0.027	<0.001

P_1_, *p*-value for trend in the number of patients, assessed by the Mann–Kendall trend test; P_2_, *p*-value for trend in the proportion of patients, assessed by the Cochran–Armitage trend analysis.

### Trends in treatment patterns

**Figure 2:**
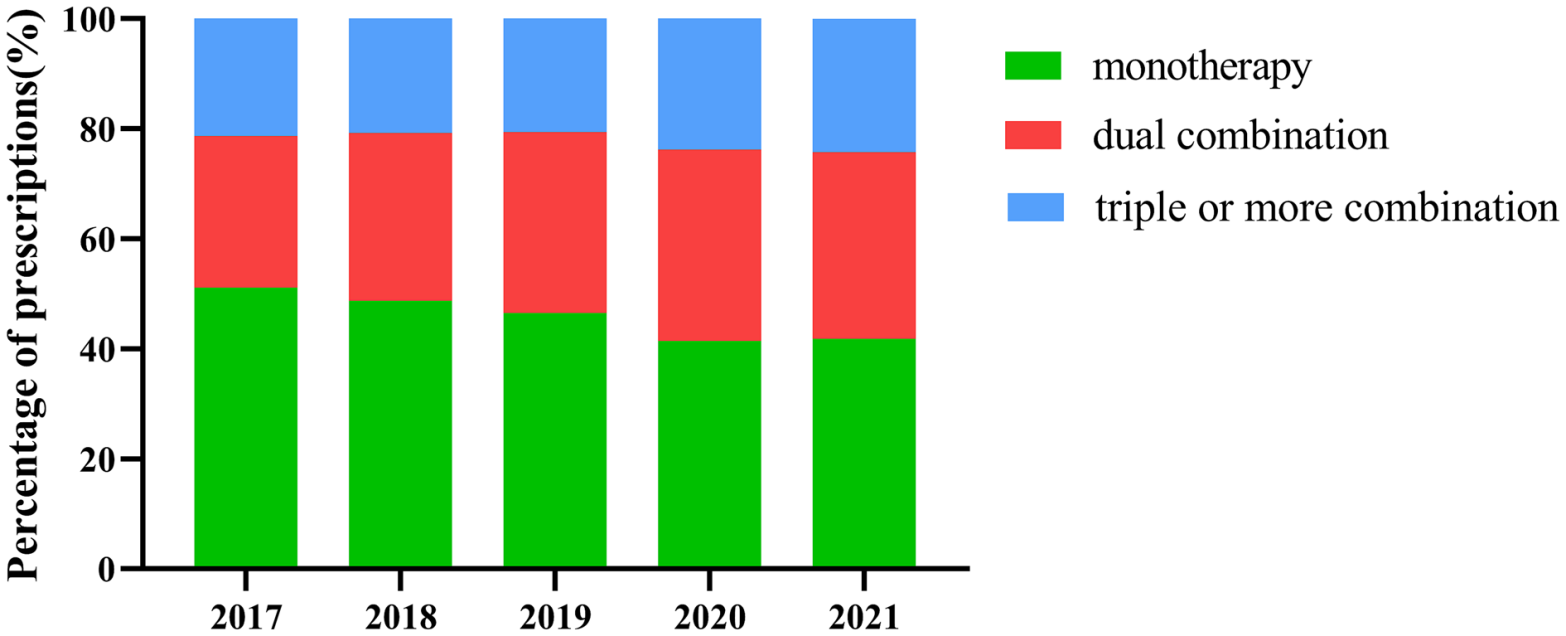
Trends in patterns of adolescent depression treatment.

Figure 2 shows the trends in treatment patterns. Combination therapy was the predominant pattern of treatment, monotherapy showed a decreasing trend over time (*p* < 0.001), while the proportion of dual combination therapy and triple or more combination therapy kept on increasing during the 5-year period (both *p* < 0.001).

### Trends by drug class

**Figure 3:**
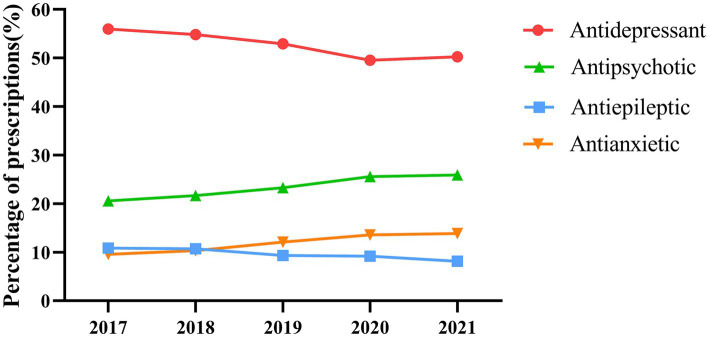
Prescription trends by drug class.

Figure 3 and Table 3 show annual prescriptions of drug classes and specific drugs. The most commonly prescribed drug classes were antidepressants, antipsychotics, antiepileptics, and antianxietics. Antidepressants remained in the leading position of the prescriptions (49.52–55.99%) though the percentage showed a decreasing trend (*p* < 0.001). The use of antipsychotics (20.55–25.89%), and anxiolytics (9.56–13.86%) increased over the period; however, the percentage of antiepileptics decreased from 10.86% in 2017 to 8.14% in 2021 (all *p* < 0.001). Nearly half of the patients were prescribed sertraline, the most commonly prescribed drug, but its use rate among visits continued to decrease (*p* < 0.001). At the end of the study, quetiapine and fluoxetine were the second and third most commonly prescribed drugs, respectively, and both showed an increasing trend (both *p* < 0.001).

**Table 3:** Prescription visits by drug and drug class, 2017–2021.

Drug class	Drug	Number of visits (%)					P_1_	P_2_
		2017	2018	2019	2020	2021		
Antidepressant	Sertraline	945 (47.90)	1,521 (50.36)	2,829 (50.63)	3,483 (46.55)	4,231 (43.39)	0.027	<0.001
	Fluoxetine	139 (7.05)	289 (9.57)	581 (10.40)	851 (11.37)	1,076 (11.03)	0.027	<0.001
	Fluvoxamine	163 (8.26)	213 (7.05)	423 (7.57)	659 (8.81)	867 (8.89)	0.027	<0.001
	Escitalopram	176 (8.92)	202 (6.69)	295 (5.28)	370 (4.94)	728 (7.47)	0.027	0.971
	Venlafaxine	118 (5.98)	126 (4.17)	158 (2.83)	158 (2.11)	223 (2.29)	0.043	<0.001
	Duloxetine	45 (2.28)	60 (1.99)	127 (2.27)	176 (2.35)	190 (1.95)	0.027	0.570
	Mirtazapine	59 (2.28)	79 (2.62)	124 (2.22)	158 (2.11)	185 (1.90)	0.027	0.016
	Agomelatine	4 (0.20)	25 (0.83)	111 (1.99)	206 (2.75)	433 (4.44)	0.027	<0.001
	Trazodone	32 (1.62)	42 (1.39)	124 (2.22)	174 (2.32)	287 (2.94)	0.027	<0.001
Antipsychotic	Olanzapine	203 (10.29)	303 (10.03)	563 (10.08)	737 (9.85)	851 (8.73)	0.027	0.036
	Quetiapine	161 (8.16)	325 (10.76)	724 (12.96)	1,155 (15.43)	1,593 (16.34)	0.027	<0.001
	Aripiprazole	125 (6.34)	166 (5.50)	338 (6.05)	488 (6.52)	685 (7.02)	0.027	0.047
	Lithium carbonate	68 (3.45)	145 (4.80)	305 (5.46)	608 (8.12)	704 (7.22)	0.027	<0.001
	Risperidone	60 (3.04)	70 (2.32)	170 (3.04)	235 (3.14)	404 (4.14)	0.027	<0.001
Antiepileptic	Sodium valproate	164 (8.31)	272 (9.01)	424 (7.59)	517 (6.91)	554 (5.68)	0.027	<0.001
	Magnesium valproate	77 (3.90)	84 (2.78)	150 (2.68)	206 (2.75)	289 (2.96)	0.027	0.492
	Lamotrigine	53 (2.69)	83 (2.75)	195 (3.49)	261 (3.49)	334 (3.43)	0.027	0.166
	Oxcarbazepine	32 (1.62)	59 (1.95)	69 (1.23)	173 (2.31)	155 (1.59)	0.086	0.822
Antianxietic	Alprazolam	135 (6.84)	223 (7.38)	439 (7.86)	805 (10.76)	844 (8.66)	0.027	0.004
	Lorazepam	75 (3.80)	151 (5.00)	252 (4.51)	479 (6.40)	648 (6.65)	0.027	<0.001
	Tandospirone	52 (2.64)	44 (1.46)	194 (3.47)	272 (3.63)	420 (4.31)	0.086	<0.001
	Oxazepam	25 (1.27)	62 (2.05)	206 (3.69)	154 (2.06)	356 (3.65)	0.086	<0.001

P_1_, *p*-value for trend in the number of patients, assessed by the Mann–Kendall trend test; P_2_, *p*-value for trend in the proportion of patients, assessed by the Cochran–Armitage trend analysis.

## Discussion

In China, there is limited research being conducted on prescription patterns for adolescents with depression. As far as we know, this study is the most comprehensive evaluation of drug use and trends among this patient group in China because the data were drawn from 136 hospitals located in nine major areas of China, and the results are nationally representative. Adolescents with depression are often accompanied by other complications. There are more than 70 diagnoses including depression in China. The objective of this study was to understand the prescribing patterns of adolescent patients diagnosed with depression in China.

As we know, the prevalence of depression increased significantly in China and around the world (2, 12). However, in this study, over the course of the study period, there were no significant trends among patients with depression. When the data from the last 2 years were excluded, it showed a significant increasing trend. The abnormal fluctuations in 2020 and 2021 may be due to COVID-19, which has spread quickly around the world since December 2019. Many studies have confirmed that outpatient and inpatient volume in healthcare facilities at all levels in China declined significantly during pandemics (13–15). It is important to note that adolescent visits have risen sharply over time, especially in female subjects, and female visits were twice as common as male visits in 2017, while the ratio jumped to 3.1 in 2021, according to several epidemiological studies (16–18), approximately twice as many females suffer from depression as males; however, the factors causing this difference to remain unclear, and biological (19, 20) and social stress (21) mechanisms might account for the gender gap.

Guidelines (22, 23) in many countries suggest a stepwise approach for the treatment of depression in adolescents. For mild depression, the National Institute for Health and Clinical Excellence (NICE) and the American Psychological Association (APA) recommend that psychotherapy and cognitive-behavioral therapy might be sufficient interventions, and antidepressant medication should not be used in the initial stage of treatment. For adolescents with moderate-to-severe depression, NICE recommends combined therapy (fluoxetine and psychological therapy) if adolescents fail to respond to a specific psychological therapy after four to six sessions, and suggests that sertraline or citalopram is the second-line treatment when fluoxetine does not work or is not tolerated due to side effects. Fluoxetine was approved by the US Food and Drug Administration (US FDA) to treat depression in children aged 8 years or older, and escitalopram was approved in adolescents aged 12–17 years. In China, none of the antidepressants was approved for treating depression in adolescents until 31 May 2021; from that day on, fluoxetine was approved by the China Food and Drug Administration (C FDA) for the treatment of moderate-to-severe depression in children aged 8 years or older. In our study, the most commonly prescribed drugs for treating adolescent depression were antidepressants. More than half of the prescriptions and more than 80% of visits used it. Selective serotonin reuptake inhibitors (SSRIs) were the predominant antidepressants in China. Among the antidepressants, sertraline occupies the most prominent position. Although its use rate continued to decrease, those that ranked second to fourth were fluoxetine, fluvoxamine, and escitalopram in 2021, whose proportions were 11.03, 8.89, and 7.47%, respectively. The substantial use of sertraline for adolescents was also reported in other countries (24–27), which may be owing to its effectiveness and well tolerated with mild adverse reactions (28, 29). Fluoxetine was the first drug approved by the US FDA for the treatment of adolescent depression, and according to previous meta-analyses (30, 31), fluoxetine (alone or in combination with cognitive-behavioral therapy) was the most effective treatment for acute moderate-to-severe depressive disorder in adolescents. While only one in ten visits used it in China, the usage of fluoxetine showed a significant increasing trend over the study period, and the latter trend is worth paying attention to. Fluvoxamine was approved for obsessive-compulsive disorder (OCD) and has a good therapeutic effect for depression with psychotic symptoms, anxiety, fear, impulse motion, suicidal behavior, and obsessive thinking. In Japan (27), fluvoxamine was the third-choice agent (selected by 13% of physicians) for adolescents with depression because of the long history of clinical use and safety profile known. Similar results were achieved in our study as the ranked third antidepressant, prescription of fluvoxamine increased progressively and the proportion maintained at approximately 8% for years. Escitalopram, the S-enantiomer of racemic citalopram, was recommended for patients older than 12 years by the US FDA, and a small amount of evidence showed that escitalopram was effective for depression in adolescents (32); however, there was just a small proportion of prescriptions for escitalopram in our study, and the high cost may restrict its use. A significant increase in suicidal behavior was observed in young people taking venlafaxine (31), and NICE guidelines (22) recommend against its usage. A downward trend of venlafaxine has been observed in this study, which is encouraging.

Depressive episodes were not only the core of depressive disorder (unipolar depression) but also the main clinical phase of bipolar disorders (BDs). BDs are psychiatric disorders with both depressive and manic episodes, which include bipolar I disorder and bipolar II disorder (33). By the end of this study, the percentage of patients receiving monotherapy was less than half and showed a significant decreasing trend over time, which suggests that the standard therapeutic approach failed to achieve satisfactory results on a majority of occasions. For adolescents with BD, augmenting the treatment strategy with antipsychotics or mood stabilizers should be considered. Lithium carbonate is classified as an antipsychotic drug in China’s current medical insurance list; therefore, in this study, we analyzed it together with other antipsychotics. Lithium carbonate as the first-generation mood stabilizer was approved by the US FDA for the treatment of manic or mixed episodes in adolescents. Atypical antipsychotics (olanzapine, quetiapine, aripiprazole, and risperidone) as second-generation mood stabilizers were also approved, while valproate (first-generation mood stabilizer) has not been approved up to now. In China, the use of sodium valproate coincides with US-FDA approval which showed a significant downward trend. Unfortunately, due to the fragmented evidence of psychotropic agents in adolescents, there was still no consensus on the efficacy of the drug on BD. For instance, Renk et al. (34) found that atypical antipsychotics were more effective than lithium in the treatment of manic and mixed episodes; however, McIntyre et al. (33) regarded lithium as the gold standard mood stabilizer. When it comes to treating a depressive episode of BD, the US FDA recommends a combination of olanzapine and fluoxetine, while according to experts in China, quetiapine, olanzapine, and a combination of lithium and lamotrigine were recommended as Class A (bipolar I disorder), and quetiapine was recommended as Class A (bipolar II disorder). In our study, quetiapine has replaced olanzapine as the most prescribed antipsychotic since 2018, which was consistent with the Chinese guidelines, and although prescriptions of lithium accounted for a small proportion of visits, its growth rate was the fastest and more than doubled in the 5 years. In addition to the efficacy, the safety of psychotropic agents needs to be considered. A high risk of weight gain was found for olanzapine and quetiapine. Risperidone and olanzapine showed significantly higher prolactin increase (35).

Antianxietic is a type of medication used to relieve anxiety and tension. Throughout the study period, the top four most used antianxietics were alprazolam, lorazepam, oxazepam, and tandospirone; the first three drugs belong to the benzodiazepines (BZDs) and tandospirone is a 5-hydroxytryptamine (5-HT) 1A receptor agonist. For the treatment of anxiety in adolescents, guidelines recommend SSRIs as first-line pharmacotherapy and serotonin-norepinephrine reuptake inhibitors (SNRIs) as the second-line pharmacotherapy (36–38). Studies of BZDs in adolescents with anxiety were limited, and some controlled trials have not shown efficacy. Simeon et al. (39) found that alprazolam and placebo did not differ statistically in terms of clinical global rating. Similarly, in another study of alprazolam (40), there were no significant differences among the alprazolam, imipramine, and placebo groups on change in anxiety scales. In this regard, the rapid rise of BZD prescriptions in China needs attention. Clinicians should use BZDs more cautiously not only because of their low efficiency but also because of the possibility of substance dependence, substance abuse, and side effects (36). Compared with the BZDs, tandospirone has a significantly lower abuse potential (41). In a study of adolescents with anxiety disorder (42), tandospirone has shown safety and efficacy that was not inferior to sertraline; however, larger and longer clinical trials are needed.

This study had some limitations. First, the cohort was not stratified by complication or severity. There are no data on how the prescription pattern differs between patients with depression alone and depressed patients with comorbidities. They require a more detailed investigation. Second, the included hospitals were all located in major cities, which may have caused sampling bias.

## Conclusion

In summary, the prescriptions and cost of treatment for adolescent depression patients both increased rapidly. The widespread use of those drugs with weak clinical evidence reflects the current state of China, which should arouse our attention. The study can provide references for clinical treatment decisions and a basis for more efficient allocation of health care resources by the government.

## Data availability statement

The raw data supporting the conclusions of this article will be made available by the authors, without undue reservation.

## Author contributions

LW and FW: conceptualization. LZ, YZ, and JY: data curation. LW, LZ, YZ, JY, NB, WF, and FW: formal analysis. FW: funding acquisition and resources. WF and FW: investigation. LZ, YZ, JY, and NB: methodology. WF and FW: validation. All authors contributed to the article and approved the submitted version.

## Footnotes

^1^ http://www.R-project.org
